# School Health Promotion at the Time of COVID-19: An Exploratory Investigation with School Leaders and Teachers

**DOI:** 10.3390/ejihpe11040087

**Published:** 2021-10-02

**Authors:** Francesca Brivio, Laura Fagnani, Simona Pezzoli, Ilenia Fontana, Luca Biffi, Alessandro Domenico Mazzaferro, Veronica Velasco, Andrea Greco

**Affiliations:** 1 Department of Human and Social Sciences, University of Bergamo, 24129 Bergamo, Italy; francesca.brivio@unibg.it (F.B.); laura.fagnani@guest.unibg.it (L.F.); simona.pezzoli@guest.unibg.it (S.P.); andrea.greco@unibg.it (A.G.); 2 Ufficio Scolastico Regionale per la Lombardia, Ambito Territoriale di Bergamo, 24121 Bergamo, Italy; uff.promozionesalute@istruzione.it; 3 Addiction Prevention Unit, Department of Hygiene and Health Prevention, Agenzia di Tutela della Salute (ATS) Bergamo, 24121 Bergamo, Italy; luca.biffi@ats-bg.it; 4 Health Promoting School Network—Bergamo, Aldo Moro School Institute, 24050 Calcinate, Italy; dirigente@iccalcinate.edu.it; 5 Department of Psychology, Milano-Bicocca University, Piazza dell’Ateneo Nuovo, 20126 Milan, Italy

**Keywords:** educational well-being, school education, health promotion school leaders, teachers, self-efficacy

## Abstract

The Coronavirus pandemic has impacted the entire school population’s emotions and the disruption of the organization of the school world. In this context it is important to reflect on the role of health promotion at school. The present study aimed at exploring school leaders’ and teachers’ perspectives and experiences about COVID-19 pandemic and its effects in the school and education system. The first objective was to gather the experience of school leaders regarding the change in school organization, with particular attention to organizational and health promotion aspects. The second was to investigate the perception of health promotion and self-efficacy of teachers in primary, middle and high schools. The research was conducted using qualitative (focus groups for the school leaders) and quantitative methods (questionnaires for the teachers). The findings showed new ways of improving wellbeing at school and implementing health promotion through the sharing of good practice between school leaders. The need for time and space to reflect among school leaders on the educational and didactic aspects of school organization also emerged. Teachers showed a low to medium level of self-efficacy regarding the adoption of strategies in line with health promotion; specificities for each grade and level will be discussed.

## 1. Introduction

To limit the spread of coronavirus disease (COVID-19), and its associated negative public health consequences, most countries in the world have decided to implement non-pharmacological interventions since March 2020. One of the most common measures used has been the closure of different types and grades of schools for certain periods during the school year, based on epidemiological trends. In Italy, namely the province of Bergamo, that is the primary city involved in the project discussed in this paper, the ongoing pandemic situation has forced periodic quarantines among the school population to contain the risk of COVID-19 virus infection.

The use of new technologies has also been increased over the past because of the need for social and physical distance. Even though people have been physically separated, they have been able to connect through social networks and the use of technological tools. As asserted also by Psacharopoulous et al. [1], the global COVID-19 pandemic has caused significant uncertainty for students and teachers and the great diffusion of media communication due to the pandemic is especially evident in this population and in the educational field. According to the United Nations Educational, Scientific and Cultural Organization (UNESCO), school closures have been impacting over 90% of the world’s student population. In many countries, distance learning has been activated by governments as a measure to maintain continuity of learning, which is characterized by the use of media and technology to enable the relationship and educational exchange between teacher and student. 

In Italy, schools were first closed on 6 March 2020; since then, there have been several schools re-openings and closures based on the contagion trend within the various Italian regions. Even though schools have been delivering education remotely in accordance with the ministerial indications, there has not been a standard model of home-based distance learning. Some schools decided to try to apply the structure of a classic frontal lesson on the web, using learning platforms; others chose web-based exchanges between class groups and teachers using social media or e-mail. As asserted by Commodari and La Rosa [2], the new coronavirus pandemic has revolutionized the school and teaching systems: all schools have had to use media and technology to continue their educational activities and relationships with students and, therefore, distance learning has become the norm. Due to the COVID-19 pandemic, an important and urgent question has emerged regarding the impact of distance education on the learning processes and psychological well-being of students, in particular children and adolescents. Undoubtedly, these are unprecedented conditions that may encourage stress and anxiety in both students and teachers.

Furthermore, it cannot be denied that equity considerations are also necessary. The pandemic has uncovered monumental inequalities in resources available to schools and families. Even though schools in better-resourced neighborhoods transitioned to online without significant barriers, schools in less-resourced ones confronted countless inequities due to limited access to devices and the internet.

In this complex scenario, it is important to ask what specific role schools could play in health promotion, given that—in a time prior to the current pandemic—the link between health promotion and schools was strong and was also the subject of much reflection. 

Health promotion in a school setting can be defined as any activity undertaken to improve and/or protect the health and well-being of the whole school community. It is a broader concept than health education and encompasses policies for a healthy school, the physical and social environment of schools and links with the local area [3,4,5].

From this perspective, and in line with the recommendations of the World Health Organization (WHO), a holistic school approach should be promoted, whereby schools take ownership of health promotion in their own context and where health is not a thematic content but an integral part of everyday teaching activity. Health and education are interconnected: by promoting health in one’s own school, it is possible both to achieve educational, social and professional goals and to promote the health of the whole school community. With this in mind, ‘Schools for Health in Europe’ (SHE network) is based on the holistic approach. It therefore orients the organization of the school community as a whole, not just the curricula, towards the promotion of healthy lifestyles, helping to create a favorable environment for students to develop the knowledge, skills and habits needed to live healthy lives into adulthood [6].

The Health Promoting School (HPS) model may offer a useful framework to develop a vision that can guide health and educational systems based on quality, equity and well-being. The Recommendations from the WHO-Europe Technical Advisory Group (TAG) for schooling during COVID-19 affirms that “the principles of health promoting schools (HPS) are even more important in a pandemic” [7]. The HPS approach acknowledges that learning and health are strictly linked, aims at individual and organizational change, recognizes that all school aspects can impact students’ health and offers health education and promotion programs and services [7]. The HPS approach has been promoted by the World Health Organization (WHO) for over 25 years and several national and international networks of schools and health institutions have been established in recent years in Europe and other continents [8,9,10]; obviously, the HPS model has been conceptualized and implemented in different ways according to professional backgrounds and contexts’ characteristics. This approach makes clear the importance of a collaboration between the education and health systems [11], which would guarantee a multidisciplinary approach, an integration of competencies and coordination in policy development. It fosters sustainable, equity-oriented, evidence-based and multilevel strategies. Nowadays, many of the HPS characteristics may be crucial during the COVID-19 pandemic. The HPS approach includes the health promotion core elements identified by international agencies to guide action during the emergency, such as intersectionality, sustainability, empowerment and public engagement, equity and a life course perspective. The HPS policies and implementation are based on intersectoral collaboration between the health and education sectors. This supports collaboration between schools and families, community and health services and community-wide endorsement [12]. Sustainability, empowerment and equity are among the HPS core values [13]. Finally, evidence-based programs and good practices also represent practical tools to support a health promotion strategy in this difficult period [14].

The Lombardy Region is one of Italy’s most populated regions and one of the first and most affected areas in Europe during the first wave of COVID-19. In Lombardy, the HPS Network was developed in 2011 through an intersectoral collaboration between the health and education system, including about 500 schools, which represent about 50% of the schools in the region. The network has involved regional and local coordinators belonging to schools, the school office and the health units. It is managed by a regional board composed of representatives from the Welfare Direction of the Regional Government, the Regional School Office and one regional head school represented by its school leader. Similar local boards coordinate the network in each province involving local health units, local school offices, and a school leader for each province. The Milano-Bicocca University—Psychology Department supports the regional board and assembly through a formal agreement offering methodological support and connections with the School for Health in Europe (SHE) Foundation. In March 2020, almost overnight, schools in many countries were forced into a drastic change from face-to-face to remote learning, in which students’ well-being and performance were tested in a way never experienced before. 

The Coronavirus pandemic subjected the entire school population to a particularly difficult situation, characterized by both the impact on emotions and the disruption of the entire organization of the school world.

Closing and reopening schools was often a major challenge and a critical step: decisions were taken in a matter of days and often the rules to be followed were unclear. Schools had to deal with the relevant and unprecedented challenges of social distancing, sanitization and careful time scan of the students’ access to the school, to avoid overcrowded classes. This led to new concerns and challenges for the school staff, primarily for school leaders and teachers. 

School leaders have been caught in the unfavorable position of being the pinch point in the system. They had to rely on others’ guidance about COVID-19 responses, processes, procedures and protocols, which could have been suddenly modified, depending on the virus development. At the same time, school leaders have been managing an unstable situation about staff resources, resulting in more things to do for the leaders. The social distancing among school staff and students has meant extra work and pressure on those staff resources who could return to work in presence. School leaders therefore felt the burden of every expectation, from above or below, that asked more commitment professionally and personally. [15] Additionally, it is likely that before COVID-19 most school leadership preparation and training programs did not keep pace with the challenges faced by schools. In many cases, existing programs and leadership models require radical rethinking and significant changes.

To date, in literature, there is little research that studies how school leaders have been responding to the pandemic, showing some emerging leadership insights within the COVID19 educational landscape [15], highlighting the significant and irreversible changes of school leadership policy due to COVID19 [16].

In addition to school leaders, teachers were among those involved in the changes due to this pandemic. Indeed, in a short time, they had to re-invent themselves and their teaching method in a way that they had never experienced or learned during their training. 

Teaching has moved from a mode where pupils and teachers shared the same physical space to a distanced one, mediated by the use of a screen and technology. This entails a major change in emotional, behavioral and organizational levels, but also in the transmission of learning.

Several international studies, researching the emotional repercussions of the COVID-19 pandemic on health professionals and vulnerable population groups in general, have been conducted. Engaging in distance learning was one of the most important changes required from teachers [17], which was influenced by the fact that students had different levels of access to online technology [18]. In addition, some teachers had to manage not only their professional role but also their parents’ one and their responsibilities towards their children’s home education, or the care of vulnerable family members.

This study bases its theoretical framework for the concept of health on what has been established by the WHO since 1948. Health is defined by the WHO as a state of complete physical, social and mental well-being, and not merely the absence of disease or infirmity [19]. This position is in continuity with the objectives of the exploratory research presented below. In fact, the objectives are built around a holistic concept of health, declined in the school field, which necessarily considers health associated with a state of well-being not only physical but also emotional and relational. 

As emerged from the literature, the following pages will describe the experiences, difficulties and changes faced by some school leaders and teachers during the specific period of the re-opening of schools. 

This exploratory research aimed to explore school leaders and teachers’ perspectives and experiences about COVID-19 pandemic and its effects on the school and education system in Italy, specifically in the province of Bergamo.

The first objective was to gather the experience of school leaders regarding the change in school organization that has taken place because of the COVID, with particular attention to organizational and health promotion aspect. The topics taken into consideration included promoting civic participation and educability; active learning and methodology; sociality and movement; fragility; exploring teachers’ perceptions of health promotion; and investigating teachers’ perceived self-efficacy. 

The focus was on sharing good practices and rethinking school organization and discuss in-depth aspects of change related to the pandemic, not only educational issues. Particularly, thoughts, analysis and sharing of the educational offer, which had been penalized by focusing on hygiene and outbreak prevention standards, were promoted. 

The second objective was to investigate the perception of health promotion and self-efficacy among primary and secondary school teachers in Italy as well as their perception of health promotion in this particular historical period, characterized by alternating teaching methods, i.e., classical face-to-face learning and DAD (distance learning).

In this perspective, the results will be used to understand which future actions may be necessary to improve the schools educational offer, focusing on the learning of self-expression in a perspective of well-being promotion.

## 2. Materials and Methods

### 2.1. Participants

It was decided to involve both school leaders and teachers as the sample for the study for two reasons: this allows for the collection of different points of view within the school and also there is currently little literature on the topic, especially that gives voice to the point of view of school leaders. The participants were recruited according to the following procedure: an invitation email was sent to the school leaders of the province of Bergamo, both to those belonging to the Health Promoting School network and to those not belonging to that network. A twofold request was made for school leaders to participate in a focus group and for teachers to participate in a quantitative-qualitative interview. A total of 19 primary and secondary school principals showed interest and availability. Of the 19 managers 11 were females (57%), 8 males (43%) with a mean age of 49.51 (MIN = 42, MAX = 59, DS = 5.75). of which 6 with less than 2 years of experience in the role. The teachers who responded to the questionnaire were a total of 66, divided into 18 from elementary school: of which 14 females (81%), 1 male (4%) and 3 not available. (15%), with a mean age of 47.63 (MIN = 38, MAX = 59, DS = 6.68), 25 from first degree secondary school of which 20 females (78%), 2 male (9%) and 3 not available (13%), with a mean age of 44.17 (MIN = 42, MIX = 47, DS = 1.94), and 23 from second degree secondary school of which 17 females (75%), 6 male (25%) with a mean age of 51.86 (MIN = 39, MAX = 66, DS = 7.76).

### 2.2. Instruments

**Figure 1:**
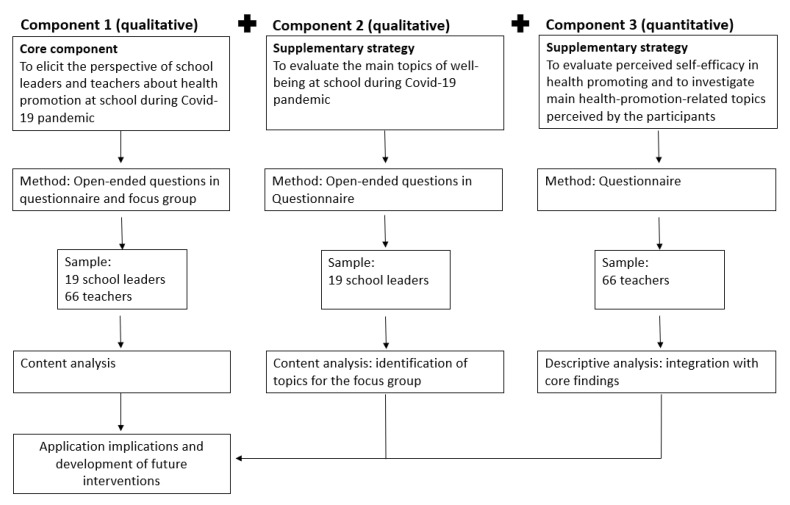
Mixed methods used in the study.

This exploratory research was conducted using a mix of methods: qualitative and quantitative data were collected at the same time, analyzed separately by the authors (Figure 1), and finally the results were compared [20,21].

The data were collected through the use of questionnaires and focus groups. Two questionnaires were administered through an online platform. A first short questionnaire was used with the school leaders to collect basic personal and professional data (name, surname, e-mail address, type of school) with some questions to stimulate an initial reflection and interest in the main topics of the subsequent focus groups (this questionnaire was created by the authors on the basis of the research objectives taking into account the current pandemic period). The questions explored the managers’ ability to reconcile the need to prevent contagion and contain the spread of the Coronavirus, with the requirement to guarantee the students’ right to education through a rich educational offer focused on their well-being. Some examples of questions that were included in the questionnaire are reported below:

We ask you to think about the health and well-being of your students. Which words come to your mind? Write down 4 verbs in the order they occur to you.

- From your point of view, how can the organization of time, space and procedures influence the learning and the conveyed educational messages?
- How can the organization influence the students’ health and well-being?

A second questionnaire was administered to the teachers and was composed of four parts. In the first part, information was gathered on the promotion of bio-psycho-social health at school in the COVID era. The items were created by the authors following the literature analysis of the documents produced by the network schools promoting health. Items were composed of close questions, with answers based on a Likert scale from 1 to 7 (not at all important = 1 very important = 7), for example: “How important do you think it is to address the following issues related to students’ health and to activate prevention/health promotion actions on these topics in this period?” [22]. In the second part, the authors examined, in the literature the construct and scales to measure self-efficacy. However, there is limited research that studies teachers’ perception of self-efficacy in engaging in professional health promotion practice [23,24]. The authors adapted some items to the research objectives. Two scales were created to assess the teachers’ perceived self-efficacy in health promotion, one for face-to-face teaching and one for distance teaching. The items were composed on close questions, with answers based on a Likert scale from 1 to 7 (not at all important = 1 very important = 7). The items included:

- Specific items for primary schools, investigating self-efficacy in face-to-face teaching (Alpha Cronbach 0.93), for example: “We ask you to think about the professional practices indicated in the table and to indicate how well you feel you are able to deal with them with your students”;
- Specific items for middle schools, investigating self-efficacy in face-to-face teaching (Alpha Cronbach 0.95), and distance teaching (Alpha Cronbach 0.96) for example “We ask you to think about the professional practices indicated in the table and to indicate how well you feel you are able to deal with them with your students, through distance learning”;
- Specific items for high school, investigating self-efficacy in distance teaching (Alpha Cronbach 0.94).

The third part of the instrument investigates the concrete needs and support that teachers want to receive from the school leader and from the local community services outside the school. Open questions were used for this section. some examples are given below: “What kind of support would you like to receive from your head teacher?”, “What kind of support would you like to receive from local authorities (associations, social and health services, local authorities, etc.)?”. In the fourth, final part, information about teachers’ status characteristics such as years of experience, and school characteristics (type of school and location), was collected with closed questions. The instrument is currently not validated due to limited sample size.

The questionnaire was distributed in an online form, and the participants could answer on a voluntary basis.

The questionnaire included both open-ended and multiple-choice questions. Some questions were differentiated according to the type of school in which the teachers worked. The differentiation of the questions was a choice of the experimenters, induced by the consideration that primary schools faced fewer hours in distance learning mode during the pandemic than secondary schools. 

Specifically, the open-ended questions investigated some crucial topics. The first is the difficulties and obstacles encountered by teachers in implementing health promotion strategies at school in this period of crisis.

The second theme concerns the methods and strategies used to promote health, both during teaching in the classroom, following COVID-19 health and safety standards and protocols, and in distance teaching.

Finally, the questions explored the kind of support that teachers want to receive, in the management of these challenges, from the school manager, local authorities and the network of schools that promote health.

The closed-ended questions investigated the health-promotion-related topics perceived by the participants as most important and the perception of self-efficacy about their teaching profession, in presence and at a distance.

Following the online administration of the questionnaire, school leaders were asked to participate in focus groups in an in-person mode. The leaders were divided into four thematic groups: promotion of citizenship and educability, learning and active methodologies, sociality and movement and fragility. 

These topics are part of the guidelines for health promoting schools. From a careful review of the literature, health promotion in a school community can include activities related to six macro areas [6]: Policies for a healthy school, Physical environment of the school, Relationships within the school community, Individual health competence, connections with partners and Health services at school. Among these macro areas, in line with the historical period characterized by the pandemic, the authors selected four themes relevant to the research [4].

For each of these topics a focus group was conducted, in order to investigate which aspects of the educational offer at school and school organization risked being particularly penalized due to hygiene and outbreak prevention regulations even if they are closely linked to learning and health.

The researchers used a semi-structured focus group guide (Table 1) which was carefully designed to encourage participants to create a moment of reflection, analysis and comparison about the proposed theme and to share good practice examples.

The outline of the focus group was the same for all groups, only the reference theme changed. The outline was divided into two different macro-areas, corresponding to the macro-objectives of the research. The first area included the common and shared definition of the theme, and aimed at analyzing several aspects, such as specific needs related to the theme, practices already implemented and possible in the future (organizational methods, procedures, policies, actions), and finally the point of view on the inclusion of students, with a major attention to frailty and inequality situations

The second area concerned the sharing of good practices, implemented in the period of the pandemic, describing in concrete terms roles, constraints, obstacles and resources, and the possibility of adapting them to other schools.

### 2.3. Procedure

The activity described in this paper took place during the 2020–2021 school year. The focus group was conducted in person in October 2020, in the main school of “the network of schools that promote health” in the province of Bergamo.

As far as school managers are concerned, 4 small groups have been constituted based on the choice of preference for one of the four proposed themes indicated in the registration form. The working groups analyzed some fundamental aspects of the educational offerings and school organization that are particularly at risk due to hygiene and outbreak prevention regulations but that are closely related to learning and health. In particular, the topics on which the researchers chose to focus on were promoting civic participation and educability; active learning and methodology; sociality and movement; fragility. 

Before beginning the focus group session and before answering the questionnaire, participants were given the information sheet describing the exploratory research nature. They then signed the informed consent form for both the questionnaire and the focus group.

As the first focus group activity, the participants introduced themselves and shared their previous personal experiences as school leaders, with reference to the current pandemic period. Then the participants were given an outline to follow during the focus group, which indicated the specific points related to the discussion topics of the group: A brief group discussion was conducted regarding each point presented by the outline. Once the activity was completed, a general discussion took place, highlighting the different points of view of the group.

Regarding the teachers, instead, the ones who expressed interest to participate were sent a link that led to the online questionnaire with open and closed questions.

### 2.4. Analysis

An analysis of the qualitative statistical profile was carried out on the data collected from the questionnaires administered to managers and teachers, and from the focus groups.

For the questionnaire administered to the teachers a descriptive analysis was conducted: minimum and maximum, mean and frequency analysis were carried out, using the SPSS statistical software; in the results section the graphs of the elaborated data will be discussed in detail.

For the qualitative analysis of the data, the method of thematic analysis was used.

The thematic analysis method consists in six phases: the first is the reading of the available data, possible transcription and identification of the first key concepts by the researcher. In the second phase the first coding takes place, in a systematic way, the data are sorted and categorized with codes, key labels. Grouping of data, according to codes in common themes, is managed in the third phase. In the fourth phase, re-read and checking the congruence between the codes and themes detected, generates a thematic map of the analyses. The fifth phase consists of defining and naming the identified themes. Finally, the sixth phase involves the production of a final report, giving examples of the data from the analysis and reiterating the objectives and questions of the research [25].

The authors, following the model in the literature, have thus carried out the thematic analysis of the focus groups and the open questions present in the questionnaires. The content analysis was carried out based on a reading grid that followed the structure of the outline. In a first step, the conductors analyzed independently the transcripts of the open-ended questions in the questionnaires and in the focus groups. Subsequently, a debriefing was organized between the conductors and the study coordinator for an initial reading and analysis of the collected data. A thematic analysis of the contents that emerged was carried out on paper and pencil, following the scheme and objectives of the focus groups. In a third step, the results of the analysis were presented and discussed again with all the authors and coordinators of the extensive project. Themes and sub-themes were generated, supported by illustrative quotes and verbal discussions.

## 3. Results

### 3.1. School Leaders: A Specific Viewpoint

Among school leaders, the creation of a network and exchange of information and best practices emerged as essential needs. These needs originated not only from the desire to continue the debate, but also from the value and uniqueness of the space the school leaders were offered: for the first time they found a space and a time to interact with each other among peers, where bureaucratic duties gave way to sharing of thoughts about the management of the schools, in the crucial period that started in March 2020, without forgetting the issues of health promotion. This time of discussion, in the form of a focus group, was highly valued by all participants.; the goal was to create an opportunity for reflection on practices, exchange and sharing of point of views, appreciation of what has been carried out so far and innovation and production of new ideas.

All these objectives were widely recognized and appreciated by the participants. The exchange of practices and thoughts has satisfied the need for sharing and debate in an open and dialogic atmosphere of exchange, also favored by the different professional experiences of each participant. Thanks to this dialogue, school leaders were able to emphasize the presence of many resources and recognize the great ability of innovation and adaptation that the schools showed in recent months.

A first goal of the meeting among school leaders was to share some educational premises and explore these issues taking into consideration both the learning and health dimensions. Secondly, the participants wanted to identify specific needs and difficulties in promoting each area. The meeting was therefore intended to be an opportunity to reflect on practices, to exchange and share thoughts, to enhance what had been carried out so far and to innovate and produce new ideas. For each theme presented below, a series of practices collected during the focus group were proposed. However, it is necessary to emphasize the importance of taking into consideration the context in which one operates. What may appear useful and functional for one school may, for several factors, be difficult to apply in another.

#### 3.1.1. Promoting Civic Participation and Educability

Regarding the subject matter of the group work, it emerged how the participants considered civic participation and educability from different points of view. 

A first topic of interest was education in being citizens, in respecting rules and in knowing and respecting the Constitution. Education to civic participation goes beyond individual teaching and represented the backbone that everyone in the school must deal with, together with the local community. The promotion of citizenship, in fact, concerns the entire life cycle and not only the school age. The participants then discussed the meaning of promoting citizenship nowadays. The theme of respect for the rules and shared responsibility within the entire educating community emerged. The promotion of civic participation and educability passes through the collaboration between teachers and school staff, which must be constantly cultivated. In addition, citizenship and educability are closely linked to the social context, the network of the local communities and the families. It is necessary to invest and seek a pact of trust with them, for the co-construction of an educational alliance. 

“From my perspective, it is important for us as school leaders to ask ourselves who is skilled at educating today, what is educability. In my opinion education in being a citizen, in respecting the rules and in knowing and respecting the Constitution” (School leader, group 1).

“It represents the backbone that all subjects within the school must deal with, together with the local community” (School leader, group 1)

However, the participants emphasized that relationships, especially with families, were not always easy. Which needs and difficulties can we identify within these issues? The Families, the local community and the schools need to recognize themselves as an educating community, within a shared responsibility and pact. It also emerged the need of children for spaces for signification and sharing about what is happening, where dialogue and listening play a central role. 

“The promotion of citizenship and educability passes through teamwork with teachers and school staff, with the network of the local communities and families, through the co-construction of an educational pact” (School leader, group 1).

The good practices mentioned in the meeting are four: the link with the, the bond between community and local services, collective action of the teaching staff and teaching for skills. The first has the objective of maintaining contact between the school manager and the students to build shared responsibility and to understand the real needs of the students; it consists periodic meetings between the school manager and the students to build alliances, to define and apply procedures.

The second practice is aimed at maintaining and fostering a rich collaboration with local entities, structuring shared projects, even in the current situation in which it is more complex to allow external actors to attend school or students to go to other spaces. It consists in the organization of weekly meetings, mostly at a distance, between the school manager, local organizations and associations.

The third aims to promote collective action, fostering teamwork, changing the image of the school manager as a manager and commander working autonomously and alone. It consists in sharing a pedagogical and educational project with all school staff, both teaching and non-teaching, through an organizational consultancy model and meetings focused on sharing thoughts and exploring topics related to learning and health, defining common priorities and working on motivation.

The four practice aims to motivate teachers and school staff in competence-based teaching. It consists in identifying an external supervisor with whom to carry out meetings with school staff in small groups. The presence of an external guide to help identify work priorities is essential to have more serenity and awareness in transmitting active learning to students.

#### 3.1.2. Learning and Active Methodologies

The participants in the working group defined learning as a dynamic theoretical, practical and experiential process that places the student at the center, as a unique individual. Active methodologies were defined as teaching strategies that put the student at the center of their own learning process. After a fruitful discussion, the group agreed that learning and active methodologies do not coincide, in an absolute way, with the curricular content.

“Learning is a dynamic theoretical, practical, and experiential process that places the student at the center, as a unique individual. Unfortunately, in recent months this issue has taken a back seat due to the need to limit COVID infections.” (School leader, group 2)

“In my opinion active methodologies as teaching strategies that place the student at the center of their own learning process.” (School leader, group 2)

The participants discussed the need to return as soon as possible to talk about learning, a topic that has taken a back seat in the recent months due to bureaucracy and focus on other aspects related to the COVID-19 emergency. They affirmed that learning should be one of the priorities for a school leader but, in recent months, they have found themselves performing unexpected tasks and actions such as measuring the size of classrooms and desks.

Participants brought out (or expressed) the need to motivate and increase the teachers’ interest in active ways of teaching that consider students as protagonists and that overcome the vision of sectorial learning by discipline. This change, however, is hindered by the environment and the society that acts in a logic of “everything and now”: many teachers often feel compelled to stick to the teaching programs, as sometimes requested also by parents, neglecting the importance of teaching by competence. Distance learning has made it more difficult to promote experiential modes of learning, even if it led to several profitable results.

“Our students are and should be more and more the protagonists of their own learning: during distance learning and lockdown they have demonstrated this, creating video research, photo albums, and other works supported by an excellent use of technology. in all this, the students have been at the center of the learning process.” (School leader, group 2).

Three best practices emerged from the discussion within this working group. The first, called “island desks”, had two goals, namely promoting collaborative work practices among students and between teachers and class groups, maintaining physical distance, and increasing the sense of autonomy, skills and responsibility, as well as learning through active methodologies. Moreover, it aimed to promote the inclusion of students with fragility. This practice consisted in arranging the desks in islands, i.e., “groups” of three. The students were seated facing the center of the group, sometimes with their backs to the teacher; every day a contact person for the working group was appointed, having the responsibility of communicating with the teacher and transmitting information and organizational tasks also towards the other two companions Within this organization students with fragility assumed the same responsibilities, sharing and collaboration as the others. The second practice, called agora, ensured the opportunity of meeting and exchange between students on different topics and promoted student participation and empowerment. This practice was based on the organization of common spaces as an agora/piazza, dedicated to a discussion between students through the placement of signs and the definition of spaces. In addition, students were co-protagonists and co-responsible in the cleaning and maintenance of these spaces. Finally, the third practice discussed within the thematic group was called “improvement goal” and was aimed to bring the student and his family to the center of the school’s attention; to define an educational pact of co-responsibility that included an individual goal for each student; to encourage an active role of the student in his educational path. During the first 2 months of the school year, teachers and students worked on identifying a personal improvement goal for each student.

This goal could be described, identified and pursued during the year. A tutor figure was identified among the teachers, to accompany the pupil and support them in achieving their personal goal. The students were invited to the usual interviews with families at the beginning of the year and all those involved signed the educational pact of co-responsibility, which also included the goal chosen by the student.

#### 3.1.3. Sociality and Movement

What emerged is how the participants considered sociality as a mutual recognition, a way of being in a relationship with others, with a verbal and non-verbal communication system, that conveys the approval or disapproval of the interlocutor. For boys and girls, it meant making friends, recognizing one’s own emotions and expressing them, both in words and with gestures. Non-verbal emotional expression (facial expressions, hugging), for health prevention’s reasons, was currently limited, but the group shared the idea that if sociality meant being in a relationship, then it was possible to find new ways to do so, building new ways to recognize the emotions of others and expressing one’s own.

“It is mutual recognition; it is a way of being in relationship with others, accompanied by a system of verbal and nonverbal communication that contributes to convey the interlocutor’s approval or disapproval.” (School leader, group 3).

“In my opinion sociality means forming bonds and making connections.” (School leader, group 3).

“Sociality means being in a relationship and then it is possible to find new ways to do so, building new ways to recognize others’ emotions and expressing your own” (School leader, group 3).

Sociality and movement today seem to be in contrast, in contrast with how they were conceived and experienced within the school context in the past. Nonetheless, the present situation presents itself as an opportunity to learn proper sociability, understood by the group as: 

“Learning to be together with greater respect for others, for their sphere and their space” (school leaders, group 3). 

The participants discussed the fact that often the contact that children sought so much risked to degenerate into pushing behavior and disrespectful attitudes. The new procedures established due to the pandemic allowed students to experience new forms of cohabitation more respectfully. For example, in the canteen, the context became quieter and more suitable to enjoy the mealtime and promote sociability.

The need for non-verbal communication emerged in a transversal way. For a teacher, having the face covered by a mask was limiting because it was not possible to fully convey recognition, approval and emotional state. In the past, the teacher would greet boys and girls with a smile or at least with an expressive facial expression, but now this was not possible; we wonder which implications this may have and how to build a new style of communication. This lack is felt most strongly in front of students with special educational needs, considering that non-verbal communication is an indispensable mean of social interaction and the fatigue of the present moment.

“In front of a child with special educational needs, I pull down my mask and smile, but I don’t do that with other children”; “How do you reject a hug from them?”; “How do you put the instructions into practice with them?” (School leaders, group 3). 

It is necessary to redefine what is meant by movement in this emergency phase, identifying all the contact forms still possible, despite the need to avoid the spread of COVID-19. Families were perceived by leaders as needing more assistance from the school, more focused on learning issues and on fears of future school closures, instead of focusing on the socialization aspects in the school context. 

In parents, as well as in teachers, it was observed the need to have informal communicative exchanges.

“Before the pandemic there were more opportunities for teachers and parents to meet each other and the school staff.; now meetings are only scheduled on Meet, the space with families is only virtual and necessarily scheduled.” (School leader, group 3).

One of the best practices identified within the working group, called “movement and territory” was aimed at ensuring opportunities of movement using the spaces in the surrounding area. It consisted of the use of local community spaces, such as municipal parks or other surrounding areas. Another good practice was to foster students’ responsibility and involvement in school organization, with the purpose of caring for the school spaces. Finally, an experimented good practice consisted in finding new forms of sociability, through the research and implementation of new forms of non-verbal communication. For example, the usefulness of using the body to concretize the distance to be maintained between students (e.g., by extending the arms) and to find new gestures and codes of communication (e.g., replace the hug with a common gesture of raising their arms at the same time) has emerged.

#### 3.1.4. Fragility

The concept of fragility emerged across the board in the different groups that addressed the topics described above. The group agreed in defining fragility as valuing uniqueness, on several levels.

“Fragility means an appreciation of the uniqueness of each student, family or teacher.” (School leader).

The themes of fragility and inclusion emerged as a priority in each group and in relation to the different themes. However, it is important to recognize the different forms of fragility through an appreciation of the uniqueness of each student, family or teacher. Which needs and difficulties have emerged in this regard? The first need emerged was how to identify and recognize its different forms. The importance of paying attention to situations in which physical distance could represent an additional difficulty emerged. Therefore, it was considered important to create spaces for meeting in person with families with fragilities, adopting the appropriate health precautions. In some cases, the need for a cultural mediator for a fruitful dialogue with parents could be useful. 

A further need concerned the showing of facial expressions to children with special educational needs, as a vehicle for communication; in this case some managers have expressed the usefulness of being able to remove the mask, while maintaining distance, to address directly to a student with special needs.in a situation of fragility. 

“It happens to me, to meet a child with special educational needs in the hallway and pull down my mask, so they can see my smile as I greet them. It’s not an attitude that strictly adheres to prevention regulations, but it’s an act of empathy and closeness that I believe is unparalleled towards a student’s fragility” (School leader, group 4).

The promotion of citizenship and educability also passed through the recognition of the fragility of teachers, students, school administrators, families and the local community. An educating community was considered as a community that enhanced the uniqueness and diversity of each member. Therefore, the need emerged to listen to and recognize the different situations, to co-construct ways of meeting, teaching and protection. 

The theme of fragility was placed at the center of attention when it came to learning and active methodologies.

“I strongly believe that open-ended competency-based instruction for all students should always be favored in order to include students and families with fragility” (School leader, group 4).

Some schools have been active in these terms also through the concretization of practices, welcoming the need to stimulate the students’ creativity and curiosity, by changing settings within the classrooms. One of the best practices identified within the working group, aimed at facilitating the inclusion of students with special educational needs and detecting and learning about situations of fragility. This practice, called reception of students, consisted of a meeting in co-presence with the teachers. This reduced the teaching time for the first few weeks but increased the resources for the management of specific needs, while waiting for the arrival of dedicated staff. An additional good practice shared within the group concerned emotions and nonverbal communications. The goal was to find new channels to express what, due to the use of masks and physical distancing, it was not possible to express through non-verbal communication. It was undoubtedly a path of recognition of emotions and appreciation of the importance of verbalization.

Finally, a good practice related to learning and technology was also implemented, with a focus on fragility. Its objectives were to promote concrete learning by focusing on knowledge and attention to the single student, to promote practical skills, to detect new interests, to break down language and social barriers and, by promoting through the practice of concreteness a greater inclusion of vulnerable students and attention to inequalities, to limit the phenomena of school drop-out.

These goals were pursued using concrete, experiential, practice-based learning modalities based on technological tools (e.g., video, robotics, platforms) and by creating ad hoc training paths for teachers to ensure the implementation of the educational programs.

These activities were particularly useful in schools with populations with a greater social and cultural fragility. In addition, students with fragility could work, overcoming limitations and educational barriers.

### 3.2. Teachers

#### 3.2.1. Perceptions of Health Promotion

The analysis of the answers to the questionnaire from teachers at all school levels, showed a lack of physical interaction opportunities; as a result, communication between students and teachers was reduced to the visual-verbal level only. This kind of communicative exchange is perceived as inadequate to promote optimal and effective interventions.

“The difficulties are related to all the physical limitations caused by the health emergency, specifically the restriction in the use of space and materials, in the impossibility of sharing, the need to stay away from each other and the inability to interact face-to-face.” (T. Primary school).

“In person: The lack of adequately large spaces for respecting interpersonal distancing. At a distance: Time. Frequent login-logout procedures between lessons have halved the number of teaching hours” (T. middle school).

“The greatest difficulty is the lack of an unstructured space in which the young people can express their hardships, challenges and discomforts, as well as the positive aspects and any positive experiences linked to the pandemic and to the physical distancing that we are all experiencing”. (T. high school).

Teachers in both middle and high schools reported difficulties related to DAD: in particular, technical problems (e.g., internet connection), the fatigue of extended screen time, the lack of time due to the switch to online teaching, the pressure caused by tight teaching programs and the risk of isolation of the most vulnerable subjects, who often could not participate in all remote activities. In the lower secondary schools, the lack of a space for emotional sharing of the difficulties linked to COVID and the difficulty of working in the classroom environment, the impossibility of engaging in socialization games or teamwork were also reported.

“The lack of direct contact with students, like it happens during DAD, makes it impossible to perceive and understand their emotions and their interests, expressed also with facial or body language. Distance cancels empathy” (T. middle school).

Despite the above-mentioned obstacles, the teachers at middle schools identified activities that engaged students through the creation of power point Canva, brochures, multimedia products, slogans, research paths and spaces for listening and emotional sharing as primary strategies for health promotion.

On the other hand, the teachers operating in the high schools preferred activities of discussion and debate starting from stimuli such as scientific material, brochures and multimedia products based on meetings and conferences with experts.

“[…] by asking the pupils to work on a product to share with others. It could be a Power-point, a brochure with Canva, any multimedia product that brings together their reflections and research. You could share stimulating videos to launch the activity and then invite to a reflection, first personal and then shared. If possible, we could work in small groups.” (T. middle school).

“Through listening to their moods, what they miss by not attending school, how they spend their days, how they manage to establish relationships at a distance. I also use tools such as a padlet, to give them the opportunity to write and share their emotions.” (T. middle school).

“Helping students to know/recognize/experience healthy lifestyles: -caring for one’s own body and mind by training and practicing appropriate physical-sportive activities; - caring for one’s diet, not only from a theoretical point of view; -reading articles/texts/documents and watching carefully chosen films/videos to promote/make one think about issues related to the pandemic and its consequences; -reinforcing life skills as a resource for coping with critical moments in everyday life.” (T. high school).

In order to improve health promotion at school during the pandemic crisis, the teachers of all schools highlighted as important and fundamental the support and creation of a sharing network with organizations outside the school: the Network of schools that promote health and/or local Institutions such as associations, social-health services, local authorities, etc.

A need to create spaces for discussion and sharing of good practices emerged, with a focus on developing a greater network between schools and colleagues from different institutes, to establish relationships of collaboration and sharing, as well as information and training meetings for teachers, students and families.

Secondary school teachers expressed, furthermore, the need to receive psychological support and more listening and emotional support from professionals.

Especially in the answers of the teachers of the middle and high schools, the necessity of a greater presence in schools of the local authorities emerged, in particular with the request for greater collaboration in taking charge of situations of fragility, in receiving operative and technical indications for the promotion of Life Skills and in sharing of material and practical tools.

The local community was therefore seen as a great source of collaboration and a fundamental presence.

“A shared platform where you can receive strategies, suggestions…” (T. Middle school)

“Being in a new and particularly difficult situation, I would expect ideas and material to engage the children and their families.” (T. Middle school).

“Meetings with experts or receiving strategies that a teacher can put in place for the students’ psycho-physical well-being” (T. High school).

“Ideas and material for practical activities, at relational, educational and didactic level—also for pupils with BES, ADHD…” (T. Primary).

“Courses that help to cope with distress for teachers who, as “helping professionals”, do not have spaces dedicated to this, and have to face bigger and bigger challenges due to both the COVID emergency and the psycho-social conditions of families, which are more and more problematic and difficult to manage” (T. Primary).

Figures that were in closer contact with the school, such as school leaders and parents, on the other hand, were perceived as present and often helpful. Teachers of all grades reported that they were already supported by their school leader and stressed the importance of receiving support, listening and motivation. More space for sharing and discussion should be created for parents in the middle school, instead.

“In my opinion, my head school leader is an understanding person and if I needed any kind of support, she would give it to me” (T. Primary).

“The school leader head teacher has done her utmost to provide teachers and pupils with a psychologist, with whom we were able to talk when we returned in September. I hope that the head teacher will continue to give importance to the various aspects of health, safety and work.” (T. Primary).

“As I am also part of the management staff, I think it is difficult to ask for any other kind of support from our head teacher, at the moment. I think that my head teacher supports me, within the limits of the restrictions and safety regulations, in all the initiatives and experiences I have with my students.” (T. High school).

“Meetings with some frequency. The lockdown experience had also put a strain on parents who were happy to ask for an opportunity to talk and discuss with each other.” (T. Middle school).

“I have sometimes involved parents indirectly through interviews about health and I think it could also work at a distance. Perhaps, charts of the class data could be produced, with which to start a discussion.” (T. Middle school).

“Involving parents in meetings at a distance is easier than in person. Promoting actions that are certainly effective for everyone, that really reach everyone is another thing” (T. Middle school).

#### 3.2.2. Perceptions of Self-Efficacy

**Figure 2:**
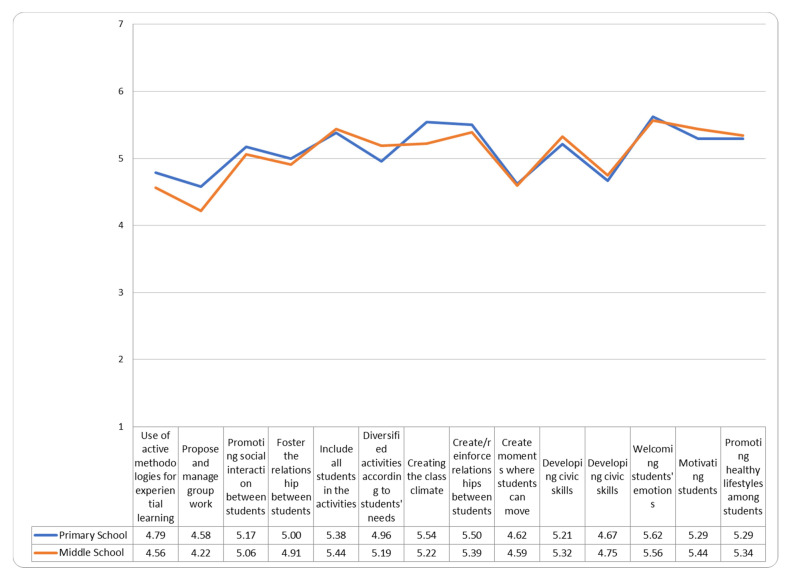
How much I feel able to address the topics listed through teaching in the presence.

The teachers of primary and secondary schools showed a low to medium perception of self-efficacy in carrying out their profession during the period of the COVID-19 pandemic. (Figure 2). In particular, they felt less able to propose group work, promote physical exercise and strengthen the students’ life skills. In carrying out their professional practices at a distance, secondary school teachers expressed a perception of medium-low self-efficacy. In this case too, the activity in which they felt they had the most difficulty was the promotion of motor activity; those in which they felt most capable were creating and strengthening the bond between teachers and students, developing citizenship skills, motivating students and encouraging healthy lifestyles.

**Figure 3:**
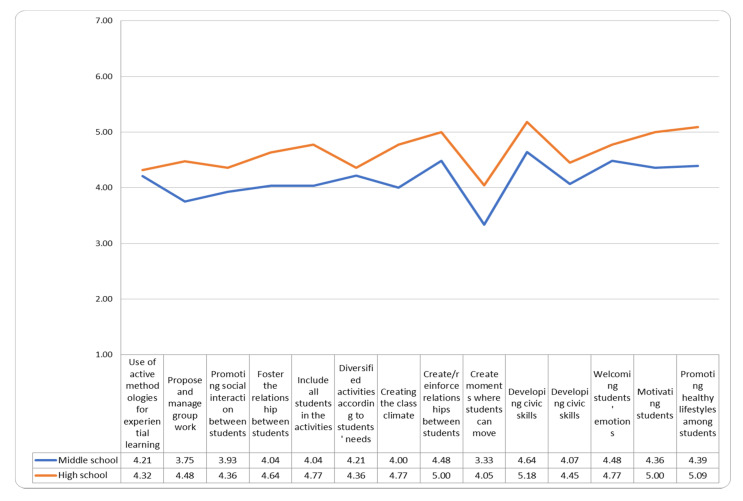
How much I feel able to address the topics listed through distance teaching.

The high school teachers, compared to the middle school teachers, felt more able to face the challenges of their professional practice, in relation to health promotion, with a distance learning mode instead of face-to-face (see Figure 3).

In particular, as we can observe from the graph, with face-to-face teaching they felt more effective in welcoming the students’ emotions, in better managing the inclusion of all the students during the activities and in creating and strengthening the bonds among the students.

**Figure 4:**
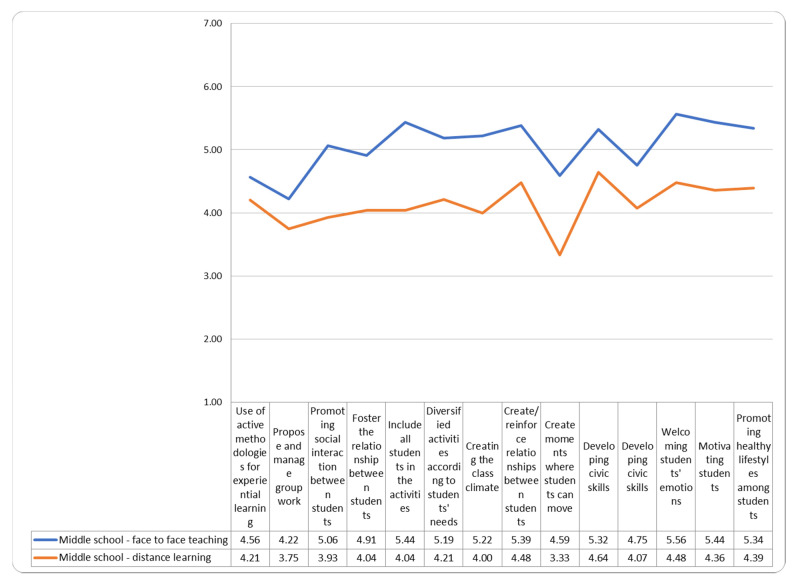
Teachers self-efficacy in the distance and face to face learning mode.

However, the perception of self-efficacy detected was at medium-low levels especially with the remote learning (see Figure 4).

Teachers were also presented with a number of health promotion topics and asked to indicate which of these topics would be most important in this particular historical period.

The elementary school teachers considered prevention/health promotion topics related to physical activity, mental health and sociality more important than addictions and hygiene in this period of pandemic crisis. The teachers working in the middle school considered important also the theme of bullying and cyberbullying, in addition to those already mentioned. Finally, the teachers operating in the high schools considered health promotion in the areas of mental health, sociality and physical activity as relevant. The topics of tobacco use, and other addictions were left out by all teachers, given the period of isolation and closures.

## 4. Discussion

This exploratory research aimed to explore school leaders and teachers’ perspectives and experiences about COVID-19 pandemic and its effects on the school and education system. The first objective was to explore the experiences of school leaders regarding the change of school organization, focusing on aspects of health promotion and the sharing of good practices. The second objective was to investigate the teachers’ self-efficacy in promoting health promotion through alternating face-to-face and distance teaching, in compliance with all COVID-19 prevention regulations. 

**Figure 5:**
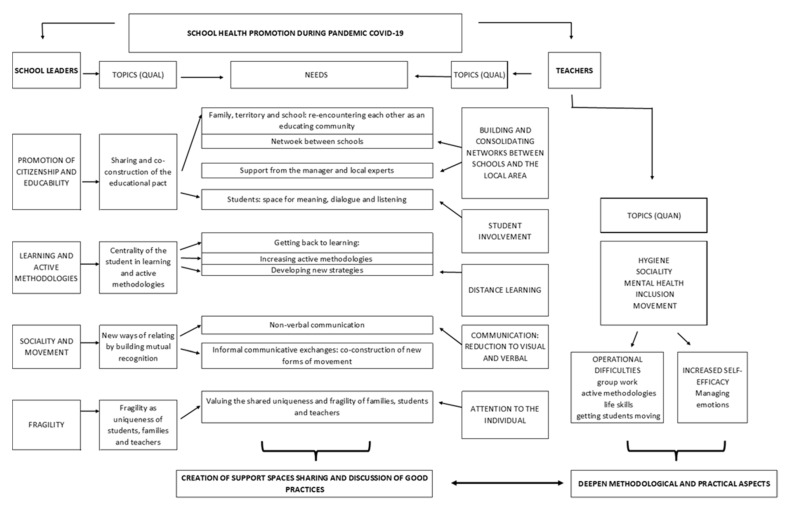
Chart of results.

The use of mixed methods allowed the integration of a qualitative and quantitative component of the results, starting from the map shown in (Figure 5) the results obtained will be discussed below.

### 4.1. School Leaders’ and Teachers’ Perspectives

School leaders and teachers consider health promotion at school to be fundamental, however, some peculiarities emerge from the research results. 

School leaders in this time of pandemic crisis spend most of their time dealing with bureaucratic issues, trying to reopen schools safely as soon as possible. Their first concern is to ensure safe teaching for all by maintaining distances and complying with hygiene rules to prevent the spread of the COVID-19 virus. Following this primary need, attention to other specific areas of health promotion sometimes takes a back seat.

Even under ideal circumstances, the contemporary headteacher role has been described as a complex, evolving and multifaceted position [26,27]. School leaders often pay a high emotional toll to lead a school during a crisis, feeling burdened by their responsibilities to serve others and putting the needs of their school community above their health and well-being [15,28]. Having highlighted the significant and irreversible changes of school leadership policy due to COVID-19, school leaders should have their self-care and self-esteem as their main priority and first concern. Indeed, leading a school through the changes and challenges, which have accompanied the pandemic, requires them to put their health and well-being first, so that they can help others. Increasingly, school leaders have been managing the emotional responses of others to this crisis, including anxiety, frustration and anger. Consequently, self-care must be a priority for school leaders at all levels [15].

Crisis and change management become essential skills for a school leader. Managing an efficient school in disruptive times requires professionals to be able to handle changes and crises, thus, it is needed the support and collaboration of all the staff. The speed of change in this pandemic has been unprecedented, so a high level of trust will be needed to ensure that problems are addressed collectively as they arise. 

Reports of 2009 about schools’ closure in England due to pandemic influenza showed that this measure was effective in controlling the spread of the disease, but little attention was given to its impact on teachers. The lack of focus and consideration on teachers is worrying because they are an essential workforce in all societies [29]. 

UNESCO report [30] showed that teachers’ confusion and stress were 2 of the 13 negative consequences of school closures, linked to the speed of closures, uncertainty about how long they will last, and unfamiliarity with distance education.

However, little research has been conducted on the emotional impact that the pandemic has had on teachers, although similar situations of closure had already occurred in some states in previous years. In these cases, the psychological effects and repercussions on learning were different, due to the lower media attention on the social and medical context [31].

### 4.2. The Importance of Networking among School Leaders

As Azorín, Harris and Jones reported [32], community and networking among school leaders, school staff and teachers is a key resource for solving problems on multiple levels, while maintaining the learning and the well-being of all those involved in school from students to professionals. 

The pandemic of COVID-19 has the potential to increase and worsen existing challenges, hence headmasters and other school leaders should acknowledge it as an opportunity to embrace new ways of thinking and change the nature of leadership and management in schools [33].

### 4.3. Utility Tools, Guidelines for Teachers

In general, the need to create spaces for comparison and sharing of good practices, especially for health promotion, emerged at all school levels. 

However, teachers reported difficulties in satisfying these needs through the shift from face-to-face teaching to distance one, as they did not feel competent in using methodologies and tools in online mode, or in ensuring an appropriate distancing and in respecting hygienic norms in face-to-face situations. 

There was a need for more networking between schools and colleagues from different institutes and sites, as well as support from experts. Primary and secondary school teachers expressed a need for more professional presence and support. In addition, secondary school teachers would have preferred to receive operational and technical indications for the promotion of Life Skills and more sharing of material and practical tools. Secondary school teachers would also have preferred to receive more listening and emotional support, as well as training and advice. 

Teachers often reported that they had attended training courses on health promotion in the past, such as life skills courses, effectiveness education, etc., during which they had been provided with much material and many guidelines for experimentation with students. During the pandemic period, and with distance learning, teachers reported that they tried to use online materials and guidelines that were provided, for example good practices to implement Life Skills Training at a distance [16] but they did not feel fully supported by external experts and local authorities. Furthermore, they were not provided with up-to-date information regarding the programs, or these were not easily accessible and usable. This led to a low perceived self-efficacy of teachers in their health promotion role. 

This is in line with the findings of a study by Velasco and colleagues [34]: health-promotion program implementation can be influenced by some aspects of the school environment; therefore, teachers require constant support and constructive and helpful appraisals from their school leader and program staff.

### 4.4. Limitations and Future Developments

This study provided detailed qualitative data. However, further research is needed to collect data from a larger sample of subjects, to confirm and extend the findings through quantitative methods. Moreover, the study presents some limitations. First, the research involved participants from a single province, limited to a single Italian region. Larger scale studies, involving the whole country or even of international scope, would provide opportunities for further investigation. However, school systems and the organization and distribution of psychosocial services differ significantly across regions and across countries, which may lead to unclear results and comparisons. Second, in terms of working with school leaders, this study is based on only one focus group carried out in-person with all the stakeholders. A further analysis articulated in multiple focus group meetings would be interesting. The third limitation is the lack of validation of the instrument for measuring self-efficacy due to the small number of subjects participating in the research. It will be the authors’ care and future objective to enlarge the sample and validate the instruments used.

However, in a context such as the one experienced in 2020, it would have been difficult to engage school leaders in additional meetings: despite their availability and motivation, they had to deal with complex school management challenges never experienced before.

Added value to the study was given by the use of mixed methods, as it allowed for a greater integration and understanding of the collected results; indeed, it has been possible to examine; on the one hand the direct experience, needs and good practices regarding health promotion, and on the other hand the direct measurement of how effective the teachers felt they were in carrying out this health promotion task at COVID-19 time. The quantitative and qualitative results of the study thus helped to complement each other by capturing features that might be omitted when using only one method.

Finally, regarding the work carried out with the teachers, a methodology that included only a qualitative-quantitative questionnaire was used.

This did not allow the collection of the teachers’ points of view, as would have been possible through a focus group. However, due to the complex school environment, in which most teachers alternated between face-to-face and distance learning activities, it would have been difficult to involve them in a more time-spending activity, even if remote.

## 5. Conclusions

Despite these limitations, this study allowed us to bring out interesting themes that are still unexplored and would need to be shared and analyzed within specially constructed spaces. 

The goal of the focus group was to gather and share good practices, with the aim of integrating two equally relevant needs: preventing contagions and containing the spread of the Coronavirus, as well as ensuring the right to education and a rich educational offer that considers the students’ well-being, personal growth and inclusion. This is a complex challenge and some major obstacles need to be considered. According to the school leaders, the main difficulties concern: the organization of spaces (e.g., the coordination of large spaces to be used by different class groups) and the timing of sanitization procedures, which in turn affect their use; the large increase in bureaucratic tasks that limit time; the impossibility of moving students from one class to another and of creating mixed groups; the experience of some teachers with the new practices and changes proposed by managers to enhance inclusive teaching and active methodologies; proposals which are perceived in some cases as invasive and limiting for learning; low availability of devices, connection and poor digital skills in some families.

Considering what has emerged and based on the feedback from the participants, it might be useful to explore some elements in further meetings, as requested by all the groups. The following are some questions related to the themes that have emerged but remained unexplored, which could be useful for future work focusing on the discussion and exchange among school leaders: how do you promote empathic learning and the use of experiential learning methods?; how do you manage school trips and movement in light of the current regulations?; how do you explain the importance of educational offerings to parents and teachers?; how to accompany teachers in changes in their teaching practices?; how to shift the focus from the learning subject to the student?; how to manage fragile students in a school with rigid spaces and movements?; and also, how to carry out future projects with the local services?; which positive aspects have been found in this new school management?; what practices could be maintained when the pandemic is resolved?

With the teachers, it is important to deepen methodological and practical aspects of health promotion through training courses with experts in the field. In particular, which tools and guidelines should be used to effectively convey aspects of sociality, inclusion and mental health to students. Which techniques and tools can be used, respecting hygienic rules and distancing or adapted to distance learning?

These questions were taken into consideration by the authors as a starting point for the development of training courses by Health Promoting School network of Bergamo.

In conclusion, the practical implications of this study concern the implementation of spaces of support, sharing and confrontation of both efforts and good practices to implement pathways for the promotion of school well-being, considering the different levels and stake holders.

## Figures and Tables

**Table 1:** Focus Group Guide.

Topic	Probes
Promotion of Citizenship and Educability/ Learning and Active Methodologies/ Sociality and Movement/ Fragility	Specific needs related to the theme
	Implemented practices (organizational arrangements, procedures, policies, actions): What has been done?
	Possible practices (organizational arrangements, procedures, policies, actions): What could be done? Which objectives and actions?
	What could be done to promote the inclusion of all students to a greater attention to frailty/inequality
	Constraints Obstacles Resources Questions and concerns
Best practices	Goal
	Description of practice
	Ways to promote inclusion/attention to fragility/inequality
	Students’ role
	Constraints Obstacles Resources Questions and concerns

## Data Availability

The data presented in this study are available on request from the corresponding author. The data are not publicly available due to privacy.
